# Independent Association of Postdoctoral Training with Subsequent Careers in Cancer Prevention

**DOI:** 10.1371/journal.pone.0144880

**Published:** 2015-12-14

**Authors:** Jessica M. Faupel-Badger, David E. Nelson, Grant Izmirlian, Katherine H. Ross, Kimberley Raue, Sophia Tsakraklides, Atsushi Miyaoka, Maura Spiegelman

**Affiliations:** 1 National Institute of General Medical Sciences, Bethesda, Maryland, United States of America; 2 Cancer Prevention Fellowship Program, Division of Cancer Prevention, National Cancer Institute, Bethesda, Maryland, United States of America; 3 Biometry Research Group, Division of Cancer Prevention, National Cancer Institute, Bethesda, Maryland, United States of America; 4 Westat Incorporated, Rockville, Maryland, United States of America; Irvine, UNITED STATES

## Abstract

The purpose of this study was to examine the career paths of alumni from the National Cancer Institute (NCI) Cancer Prevention Fellowship Program (CPFP), a structured in-house postdoctoral training program of 3–4 years duration, and specifically what proportion of the alumni were currently performing cancer prevention-related activities. The analyses here included 119 CPFP alumni and 85 unsuccessful CPFP applicants, all of whom completed postdoctoral training between 1987–2011 and are currently employed. Postdoctoral training experiences and current career outcomes data were collected via online surveys. Differences between groups were assessed using chi-square and Fisher’s exact test p-values and subsequent regression analyses adjusted for differences between the groups. Compared to 15.3% of unsuccessful CPFP applicants, 52.1% of CPFP alumni (odds ratio [OR] = 4.99, 95% confidence interval [95% CI): 1.91–13.0) were currently spending the majority of their time working in cancer prevention. Among those doing any cancer prevention-focused work, 54.3% of CPFP alumni spent the majority of their time performing cancer prevention research activities when compared to 25.5% of unsuccessful applicants (OR = 4.26, 95% CI: 1.38–13.2). In addition to the independent effect of the NCI CPFP, scientific discipline, and employment sector were also associated with currently working in cancer prevention and involvement in cancer prevention research-related activities. These results from a structured postdoctoral training program are relevant not only to the cancer prevention community but also to those interested in evaluating alignment of postdoctoral training programs with available and desired career paths more broadly.

## Introduction

It is estimated there will be more than 580,000 deaths in the United States from cancer in 2015 [1], and cancer is the second leading cause of death in the country [2]. With the aging of the population in the U.S. and elsewhere, the burden of cancer is expected to grow substantially over the next several decades because the vast majority of cancers occur among persons aged 55 years or older [1]. This makes preventing cancer a major public health priority [3]. There are several proven approaches to reduce cancer mortality and morbidity [3–6], but one issue receiving little attention is developing and sustaining a sufficient workforce trained in cancer prevention research and practice [7]. Such a workforce can help expand the discovery, implementation, and dissemination of cancer prevention measures, which range from strengthening our understanding of cancer biology and identifying new agents and interventions, to increasing the adoption of proven strategies to reduce cancer incidence [7].

The National Cancer Institute (NCI) and others have funded postdoctoral training programs in cancer prevention for more than 30 years in the United States [8–10]. To our knowledge, however, there have been no studies assessing if scientists who received postdoctoral training in cancer prevention continue to pursue careers in this field. There is a general lack of data about those who have received postdoctoral research training in the biomedical fields and their ultimate career outcomes [11–13], and some issues specific to the field of cancer prevention [7,8] make it particularly difficult to assess the alignment of training with subsequent careers in this area. These include the lack of credentialing bodies, differing definitions of cancer prevention and the diversity of disciplines and practice settings in the field [7,14]. The state of the job market in cancer prevention is also largely unknown.

We recently conducted surveys of scientists who completed postdoctoral training during 1987–2011 in the NCI Cancer Prevention Fellowship Program (CPFP). The purposes of collecting these data were to: 1) examine the extent to which scientists who had conducted cancer prevention-related research during their postdoctoral training were currently working in the field of cancer prevention in any capacity; 2) assess the extent of cancer prevention research activities among those currently working in cancer prevention; and 3) better understand the reasons why persons who previously conducted research relevant to cancer prevention were not working in this field.

In addition to determining the alignment of postdoctoral training with cancer prevention-related career outcomes, the rigorous evaluation design employed here may be of interest to the broader postdoctoral training community. To date, evaluations of structured, on-site postdoctoral training programs have been limited and have focused mostly on satisfaction measures [15]. Those evaluations reporting current employer or other career information have not included a comparison group to provide additional context for understanding the added value of the training program. Here, we also included applicants to the CPFP program to explore the career outcomes of another group with research interests in common with the CPFP alumni and use regression analysis to adjust for differences between the groups so independent effects of training on cancer prevention related career outcomes could be determined.

## Methods

### Study populations

The population of interest for this study consisted of individuals who were CPFP alumni. The structure of the NCI CPFP has been described elsewhere [10,16]. Briefly, the CPFP recruits 10–15 individuals annually through a competitive selection process to conduct their postdoctoral research in cancer prevention at the NCI facilities located in Maryland. In addition to the mentored training experience, NCI Cancer Prevention Fellows are supported to attain a Master of Public Health degree in the first year of the program (unless they already have the degree or another relevant advanced degree in a field such as epidemiology), and throughout their fellowship to receive additional scientific training focused on cancer prevention-related topics, and on leadership. NCI Cancer Prevention Fellows receive up to four years of support for their postdoctoral training through this program.

Eligible CPFP alumni participants were those who entered the program as of August 31, 1987, were fellows for at least 12 months, and left the program no later than December 31, 2011. Of the 211 who met the inclusion criteria, six were excluded (5 deceased, 1 involved in the study design), leaving a total sampling frame of 205 alumni.

The comparison population consisted of all applicants to the CPFP who were invited for an in-person interview but ultimately not selected for the program during the same time frame from which the alumni were selected, i.e., unsuccessful applicants. Scientists who interviewed for the CPFP but were not ultimately selected were chosen as the most comparable group with which to compare outcomes since these individuals shared an interest in the same training program as the alumni and had applications that were competitive for garnering an in-person interview with the selection committee. Restricting the applicant pool to those who were invited for an in-person interview, rather than using the total applicant pool, was done to minimize the potential effects of selection bias when evaluating career development programs [17]. Since these analyses focus on outcomes of postdoctoral training, all applicants for this study must have completed at least one postdoctoral training experience. Of the 281 applicants who met the eligibility criteria, a total of 22 were excluded because they were deceased or were offered a fellowship position but declined, leaving a total sample frame of 259 applicants.

Details about career outcomes from another subset of individuals (n = 143) who competitively funded their postdoctoral training from 1987–2011 through Ruth L. Kirschstein National Research Service Award (NRSA/F32) individual postdoctoral fellowships (http://www.cancer.gov/grants-training/training/funding/f32) and whose projects were related to cancer prevention, are included in the S2 File. Because this population differed substantially from CPFP alumni and unsuccessful CPFP applicants, they were not included in our main analyses.

### Development and administration of surveys

The survey instrument was developed after conducting a literature search of training program evaluations to identify questions used in previous studies assessing postdoctoral training program outcomes [15]. Key themes and questions of interest to NCI, as well as input from previously conducted in-depth interviews of alumni [16] were incorporated into the development of the survey (S1 File). The survey instrument collected demographic information such as age, gender, race/ethnicity, current scientific discipline(s), current employer, and length of time in current position. Career activities of interest spanned a variety of areas and included leadership opportunities, community service, professional activities, peer recognition, and, of particular interest to the NCI Cancer Prevention Fellowship Program, employment involving cancer prevention-related activities. The latter career area of concentration is explored in-depth here.

All participant survey activities were conducted by Westat, Inc. Initial contact information for CPFP alumni and unsuccessful applicants was gathered using CPFP database sources. If this information was outdated, Westat staff attempted to locate more current information using Internet search tools and other methods. All surveys were conducted using email with a link to an on-line survey instrument. Potential participants were initially sent an email notifying them about the survey, followed two weeks later with a mailed letter with more detailed information. Subsequent reminder emails (maximum of three) and telephone calls (maximum of two) for nonparticipants were performed at regular intervals during the data collection period from February-June 2014.

Surveys were completed by 123 CPFP alumni (59% response rate) and 115 CPFP applicants (43% response rate). The two main reasons for non-response were failure to locate respondents and unwillingness of respondents to participate. Based on the information available for comparison (i.e., applicant year and age for CPFP alumni and unsuccessful applicants), there were no significant differences between the respondents and non-respondents (p<0.05 based on chi-square tests). Furthermore, among respondents, there was no apparent overlap of individuals among the two groups. No individual identifier information was included in the final dataset, and specific demographic categories were irreversibly combined (e.g., race other than white) in cases of small cell sizes that might lead to possible respondent identification. Both the NIH and Westat Institutional Review Boards independently reviewed the study design and materials and found the study to be exempt under rule 45 CFR 46.101(b) (2).

### Definition and coding of career outcome measures

The extent of current work in cancer prevention was assessed with the question “Approximately what percentage of your current work is done in cancer prevention and control?” [Note: respondents were instructed to exclude time spent on cancer treatment or cancer treatment research.] Potential responses were none (0%), a small percentage (1%-25%), a moderate percentage (26%-50%), a large percentage (51%-75%), or a very large percentage (76%-100%). Those who reported doing any cancer prevention work were asked the following question about their current cancer prevention research activities: “Approximately what percentage of your time in your current job is spent on research and research support activities in cancer prevention and control? Please include time spent conducting research yourself, as well as time spent supporting the research of others through activities such as research management, monitoring, reviewing, funding, analysis, dissemination, and other research support activities.” Potential responses were the same as for the previous question.

Participants not currently working in cancer prevention were asked: “What are the reasons you are not currently working in the field of cancer prevention or control?” Potential responses included: a suitable job in the field was not available; a better opportunity was available outside the field; career or professional interests changed; personal reasons; or some other reason. Respondents had the option to select more than one answer.

### Statistical analysis

First, distributions of age (<40, 40–49, ≥50 years), sex, race/ethnicity (white, non-Hispanic and other than white), cohort (year most recent postdoctoral training completed, 1987–1996, 1997–2001, 2002–2006, and 2007–2011), degree type (PhD vs. other), discipline (biological science, epidemiology/public health, behavioral science, other), length of employment (<5, 5–9, ≥10 years), and employment sector (university/research center, government, other), were tabulated according to survey group. Differences in percent distributions among the population groups were assessed with chi-square or Fisher’s exact tests.

Crude association between each of the two cancer prevention effort variables, e.g., percent of current effort in cancer prevention, and percent of effort devoted to cancer prevention research work (0%, 1%-50%, or ≥51%), and survey group were tabulated. Each of the cancer prevention effort variables were then analyzed separately as the dichotomous (i.e., more than 50% compared to 50% or less) dependent variable in multiple logistic regression against the main study variable, survey group. The models were adjusted for all factors in Table 1 where there were significant differences among groups (age, sex, race/ethnicity, cohort, degree type, discipline, length of time in current employment, and employment sector). The discipline variables, which consisted of eight separate binary responses and allowed for overlap, were summarized via principal components. Loadings from the first principal component indicated that two indicators would suffice: Social Science/Epidemiology, and Biomedical Research. Initially, all analyses incorporated adjustment for non-response via inverse weighting with error estimates computed via the sandwich estimate. However, since there was little variability in the response weights versus age groups, and corresponding point estimates and standard errors were affected very little, the resulting sample was treated as a random sample. R version 3.1.2 [18], was used for all analyses.

**Table 1:** Demographic and Other Characteristics of the CPFP Alumni and Applicants.

	Fellowship Alumni (%) N = 119	Fellowship Applicants (%) N = 85	p value
**Age (yrs)**			
<40	27.9	26.5	
40–49	41.4	18.1	
≥50	30.6	55.4	<0.001
**Sex**			
Male	25.8	28.4	
Female	74.2	71.6	0.680
**Race/Ethnicity**			
White, non-Hispanic	79.5	67.1	
Other	20.5	32.9	0.046
**Cohort**			
1987–1996	20.3	31.5	
1997–2001	28.5	16.9	
2002–2006	37.4	20.2	
2007–2011	13.8	31.5	<0.001
**Doctoral Degree**			
PhD	87.7	76.5	
Other	12.3	23.5	0.036
**Discipline** ^a^			
Biological science	16.3	30.3	0.018
Epidemiology ^b^	55.3	39.3	0.024
Behavior/social science	25.2	13.5	0.041
Medicine	11.4	19.1	0.125
Nutrition science	13.8	7.9	0.190
Physical science	0.8	0.0	1.000
Mathematics	3.3	1.1	0.404
Other	16.3	20.2	0.474
**No Disciplines**			
One	67.5	75.3	
More than one	32.5	24.7	0.227
**Employment**			
Unemployed	3.25	4.49	
Currently employed	96.7	95.5	0.647
**Current Employer**			
University	32.8	52.9	
Government: NIH	39.5	10.6	
Private Company	5.0	12.9	
Government: Other	10.1	17.6	
Research center	3.4	2.4	
Self-employed	5.0	2.4	
Health clinic/hospital	1.7	0.0	
Foundation/association	2.5	1.2	<0.001
**Years in Current Job**			
<5	45.4	48.2	
5–9	32.8	25.9	
≥10	21.8	25.9	0.268

^a^ Column percentages >100 because respondents could select more than one discipline

^b^ Includes public health

NIH = National Institutes of Health

## Results

The CPFP alumni and applicants differed significantly from each other by demographics and other characteristics, with the exception of gender, some scientific disciplines, and number of years in current job (Table 1). Fellowship alumni were slightly younger and more likely to have a PhD degree than applicants. Fellowship alumni also were more likely to be in an epidemiology or behavioral/social science discipline and to work for a government agency than unsuccessful fellowship applicants.

Bivariate results on current work involving cancer prevention are shown in Table 2. There were statistically significant differences among the two populations, with 52.1% of fellowship alumni and 15.3% of fellowship applicants reporting the majority of their current work involved cancer prevention. Of those currently working in cancer prevention, 54.3% of fellowship alumni and 25.5% of fellowship applicants spent the majority of their time involved in cancer prevention research or research-related activities.

**Table 2:** Time Spent Performing Cancer Prevention-Related Work or Cancer Prevention Research.

	**Fellowship Alumni n = 114 (%)**	**Fellowship Applicants n = 78 (%)**	**p value**
**Any Cancer Prevention Work**			
None (0%)	11.8	44.7	
Some (1–50%)	36.1	40.0	
Majority (≥51%)	52.1	15.3	<0.001
	**Fellowship Alumni n = 105 (%)**	**Fellowship Applicants n = 47 (%)**	**p value**
**Cancer Prevention Research** ^a^			
None (0%)	3.8	10.6	
Some (1–50%)	41.9	63.8	
Majority (≥51%)	54.3	25.5	0.001

^a^Individuals who answered “None” to the question about any cancer prevention-related activities in their current work skipped over the question focused specifically on cancer prevention research-related activities.

Results from regression analyses on the majority of time spent on cancer prevention-related activities broadly or on cancer prevention research in respondents’ current work are provided in Table 3. The odds ratios reported in Table 3 are for the independent association of each variable with either currently working in cancer prevention or conducting cancer prevention research, after adjusting for all other variables in the table. Fellowship alumni were significantly more likely to spend the majority of their time working in cancer prevention than fellowship applicants (OR = 4.99). Reporting an epidemiology or behavioral/social science discipline (OR = 4.31) or being employed at a university/research center or government agency (OR = 6.79 and 7.40, respectively) also were independently associated with the majority of time conducting cancer prevention related work. The only statistically significant factors associated with cancer prevention research were being a fellowship alumnus (OR = 4.26) or working in a government or university setting (OR = 6.33 and OR = 7.56, respectively).

**Table 3:** Odds Ratios for Factors Associated with the Majority of Current Work Involving Cancer Prevention.

	Cancer Prevention Work Odds Ratio (95% CI)	Cancer Prevention Research Odds Ratio (95% CI)
**Population Group**		
Fellowship Applicants	1.00 (referent)	1.00 (referent)
Fellowship Alumni	4.99 (1.91–13.0)	4.26 (1.38–13.2)
**Age (yrs)**		
<40	1.00 (referent)	1.00 (referent)
40–49	0.77 (0.26–2.19)	0.61 (0.19–1.99)
≥50	0.56 (0.12–2.52)	0.54 (0.11–2.68)
**Sex**		
Male	1.00 (referent)	1.00 (referent)
Female	0.41 (0.13–1.31)	1.09 (0.33–3.62)
**Race/Ethnicity**		
White, non-Hispanic	1.00 (referent)	1.00 (referent)
Other	0.87 (0.34–2.26)	1.15 (0.41–3.20)
**Cohort**		
1987–1996	1.00 (referent)	1.00 (referent)
1997–2001	1.14 (0.28–4.67)	0.99 (0.22–4.56)
2002–2006	1.37 (0.27–7.01)	1.21 (0.22–6.79)
2007–2011	2.18 (0.37–12.7)	2.91 (0.43–19.6)
**Doctoral Degree**		
PhD	1.00 (referent)	1.00 (referent)
Other	0.29 (0.07–1.18)	0.27 (0.06–1.21)
**Epidemiology or Social/Behavioral Science**		
No	1.00 (referent)	1.00 (referent)
Yes	4.31 (1.67–11.2)	2.67 (0.97–7.36)
**Biomedicine**		
No	1.00 (referent)	1.00 (referent)
Yes	0.65 (0.23–1.86)	1.09 (0.36–3.27)
**Employer**		
Other	1.00 (referent)	1.00 (referent)
Government	6.79 (1.58–29.1)	6.33 (1.37–29.4)
University/research center	7.40 (1.65–33.2)	7.56 (1.56–36.6)
**No. years in current job**		
<5	1.00 (referent)	1.00 (referent)
5–9	1.05 (0.40–2.76)	2.14 (0.73–6.30)
≥10	1.38 (0.40–4.77)	1.99 (0.48–8.30)

The most commonly mentioned reasons by those not working in cancer prevention were a change in career or professional interests, a better opportunity in another field, or the absence of an available cancer prevention position (Table 4). When examined by population, statistically significant differences were found for change in career or professional interests, which was more often mentioned by fellowship applicants, and some other reason, which was more often mentioned by fellowship alumni.

**Table 4:** Reasons for Not Currently Working in Cancer Prevention.

	Fellowship Alumni n = 14 (%)	Fellowship Applicants n = 38 (%)	p value
Career or professional interests changed	14.3	57.9	0.005
Better opportunity in another field	50.0	42.1	0.611
Job in cancer prevention unavailable	28.6	26.3	1.000
Personal reasons	7.1	5.3	1.000
Other	28.6	5.3	0.038

Column percentages add to >100% because respondents could select more than one response.

## Discussion

These results provide an overview of the characteristics of biomedical scientists who completed postdoctoral research training related to cancer prevention at the National Cancer Institute and the extent to which they continue to participate in cancer prevention-related activities. Overall, CPFP alumni were much more likely than CPFP applicants to have cancer prevention-related work, and for cancer prevention research to be a major focus in their current position.

To our knowledge, this is the first study to demonstrate a strong and independent association between postdoctoral training in cancer prevention with subsequent employment involving cancer prevention-related activities. Inclusion of unsuccessful applicants to CPFP alumni who completed postdoctoral training elsewhere, and the use of multivariate modeling to adjust for a number of other factors, provides reassurance that this association was not likely due to chance. No comparison of individuals participating in training programs can be ideally matched to those not participating in such programs because of issues such as not selecting individuals at random and differences in subsequent training [17]. Nonetheless, unsuccessful CPFP applicants were as similar to CPFP alumni as possible in that they expressed an interest in participating in a well-defined training program dedicated to cancer prevention, and had applications judged to be competitive enough to warrant an in-person interview as part of the CPFP selection process.

Several other factors were also associated with currently working in cancer prevention. Those in the disciplines of epidemiology, behavioral science, or social science were more much more likely to perform cancer prevention work in general and cancer prevention research in particular. Reasons for this may include the greater availability of cancer prevention positions for persons in these disciplines, or for CPFP alumni in particular, additional knowledge and experience about prevention gained from earning an M.P.H. degree. Employment in a government or academic setting was associated with a higher likelihood of currently working in the field of cancer prevention. This may suggest there are more cancer prevention-related positions available within these employment sectors. It is interesting that CPFP alumni were more likely to be employed in a government setting; this may be due to self-selection of alumni who prefer to work in this sector, or because training in a government agency provides a greater awareness of the career options and types of activities associated with employment in this career sector.

There were several study limitations. Data were based on self-reports without independent verification. Survey response rates were good for an email-administered web survey [19,20], but it is possible there was nonresponse bias because of differences between survey participants and non-participants besides age and year of application. Another factor that may have influenced the analysis was that all disciplines represented among the populations here may not have equal opportunity or time to conduct cancer prevention research. For example, it is possible that the physicians in these populations would not be able to have the majority of their time involve cancer prevention research if they had extensive clinical responsibilities.

The CPFP alumni also may have a broader interpretation of, and a stronger identification with, the field of cancer prevention than the unsuccessful applicants. As noted in a previous study focused on in-depth interviews with CPFP alumni [16], many facets of the structure of the CPFP promote identification with this field. The participation in this program with a cohort of peers from a variety of scientific disciplines, multiple mentor structure, and defined curriculum components may all provide alumni with a broader context with which to assess the type of work or research that falls within the context of cancer prevention. It may also be expected that applicants not selected for the NCI CPFP could have different career trajectories by virtue of missing out on training and networking opportunities available to CPFP alumni. However, it should be noted that there were many other cancer prevention postdoctoral training programs during the time period of the study.

The study design and results presented here have implications not only for the field of cancer prevention but also present an example of how to address some of the broader issues regarding understanding the career outcomes of others who complete postdoctoral research training in the biomedical sciences. An earlier literature review of evaluation studies of structured postgraduate training programs revealed that most focused only on alumni satisfaction with the training program [15], with a few also including career outcomes, such as current job title and/or total scholarly publications. Almost without exception, these evaluations followed only the training program alumni and did not include other groups for possible comparisons. This is a major limitation as there is no benchmark, even if imperfect, for determining whether the specific training program provided an added value to the postdoctoral fellow. Although these evaluations may report outcomes, they leave the reader with the question, “Compared to what?” We have tried to provide a context for addressing that question, and examples of how to try to separate out training program-specific associations among different populations.

Recent reports about postdoctoral fellows in the biomedical sciences have expressed concern about the employment options currently available to these individuals [21,22]. But this was not the situation for CPFP alumni, with nearly 90% currently working in positions where at least some of their work involved cancer prevention. And, among those not working in cancer prevention, the vast majority had another reason besides the absence of an available position in this field.

Drawing firm conclusions is difficult because there are no estimates of the cancer prevention workforce [7], but when including a wide variety of career sectors and employment opportunities, our results suggest there is an alignment of postdoctoral training in cancer prevention with subsequent employment in this field. There are many factors that could explain these associations, but these results highlight the value in assessing career outcomes across individual programs and in specific scientific fields, in addition to considering national trends, so that prospective postdoctoral trainees can make better informed decisions about their training and career paths [11,13].

Understanding the outcomes of alumni from the NCI CPFP was the impetus for this larger evaluation effort. Therefore these analyses focused specifically on assessing the extent to which completing of postdoctoral training involving cancer prevention research was associated with a subsequent career involving cancer prevention-related work and research activities. However, additional data collected in the course of this study will also permit a closer look at postdoctoral training outcomes more broadly. In subsequent analyses, other career outcomes such as salary, leadership opportunities, and career satisfaction will be assessed across all populations surveyed. These subsequent analyses will address an identified need for the collection and reporting of data on postdoctoral training outcomes to increase the transparency of this career path [11,13,22], while also continuing to use this rigorous evaluation design to examine these data in the context of different training experiences.

## Supporting Information

S1 File Survey Instrument. Complete web survey instrument for all three populations.
(PDF)

S2 File Description of NRSA/F32 awardee subset and Supplemental Tables A-C. (PDF)
