# Supplementary material for: Independent Association of Postdoctoral Training with Subsequent Careers in Cancer Prevention
Source: PLoS One. 2015 Dec 14;10(12):e0144880. doi: 10.1371/journal.pone.0144880 (PMC4682206; doi:10.1371/journal.pone.0144880)
Supplement: S1 File — Complete web survey instrument for all three populations. (PDF) [file pone.0144880.s001.pdf]

Supplemental Material – Full survey sent to CPFP alumni (some program-specific questions were modified for unsuccessful applicants and NRSA/F32 awardees)

**EVALUATION OF THE NATIONAL CANCER INSTITUTE'S
CANCER PREVENTION FELLOWSHIP PROGRAM
SURVEY OF ALUMNI**

The National Cancer Institute (NCI) has contracted with Westat, an independent research firm located in Rockville, Maryland, to conduct a comprehensive evaluation of the NCI's Cancer Prevention Fellowship Program (CPFP). The purpose of the evaluation is to collect information on the careers and experiences of former fellows, and to get suggestions for improving the program.

Participation in the survey is voluntary and the information you provide will be kept private to the extent permitted by law. The survey data will be collected by Westat, and your individual responses to the survey, or any potentially personal identifying information, will not be shared with NCI staff members. The information collected will be published in aggregate form only and will not identify individuals in any reports or presentations. Your participation will help provide valuable information that will assist CPFP in making decisions about future program initiatives to improve postdoctoral training.

We anticipate the survey will take approximately 25 minutes to complete.

If you have any questions or comments, please contact Kimberley Raue at Westat at
(800) 937-8281, ext. 3865 or CPFPsurvey@westat.com.

OMB No: 0925-XXXX
Expiration Date: xx/xx/20xx

Collection of this information is authorized by The Public Health Service Act, Section 411
(42 USC 285a). Rights of study participants are protected by The Privacy Act of 1974. Participation is voluntary, and there are no penalties for not participating or withdrawing from the study at any time. Refusal to participate will not affect your benefits in any way. The information collected in this study will be kept private to the extent provided by law. Names and other identifiers will not appear in any report of the study. Information provided will be combined for all study participants and reported as summaries. You are being contacted by email to complete this instrument so that we can evaluate the Cancer Prevention Fellowship Program.

Public reporting burden for this collection of information is estimated to average 25 minutes per response, including the time for reviewing instructions, searching existing data sources, gathering and maintaining the data needed, and completing and reviewing the collection of information. **An agency may not conduct or sponsor, and a person is not required to respond to, a collection of information unless it displays a currently valid OMB control number.** Send comments regarding this burden estimate or any other aspect of this collection of information, including suggestions for reducing this burden, to NIH, Project Clearance Branch, 6705 Rockledge Drive, MSC 7974, Bethesda, MD 20892-7974, ATTN: PRA (0925-XXXX). Do not return the completed form to this address.

EMPLOYMENT INFORMATION

The first section of the survey asks questions about your work history and the type of work you are currently doing.

1. For the following question, please include your participation in the Cancer Prevention Fellowship Program. Which statement represents your postdoctoral or fellowship experience? *(Please select one answer.)*

   Completed **one** postdoctoral or fellowship program or position .... ☐ 1
   Completed **multiple** postdoctoral or fellowship programs or positions .... ☐ 2

2. In what year did you complete your most recent postdoctoral or fellowship position?

   ☐☐☐☐

3. Do you **currently** hold a postdoctoral or fellowship position?

   Yes .... ☐ 1
   No.. ☐ 2

4. Including self-employment, what is your **current** employment status? *(Please select one answer.)*

   Employed full-time .... ☐ 1
   Employed part-time... ☐ 2
   Retired... ☐ 3 *(Go to question 16.)*
   Not currently employed....................................... ☐ 4 *(Go to question 16.)*

5. Which **one** of the following best describes your **primary** employer? *(Please select one answer.)*

   National Cancer Institute (NCI).......................... ☐ 1 *(Go to question 8.)*
   National Institutes of Health (NIH) other than NCI.. ☐ 2 *(Go to question 8.)*
   Government agency other than NIH .... ☐ 3 *(Go to question 7.)*
   University or some other academic institution..... ☐ 4 *(Go to question 6.)*
   Independent cancer research center or some other health research institution .... ☐ 5 *(Go to question 7.)*
   Health care clinic or hospital............................... ☐ 6 *(Go to question 7.)*
   A foundation or professional association............. ☐ 7 *(Go to question 7.)*
   Private company.. ☐ 8 *(Go to question 7.)*
   Self-employed .... ☐ 9 *(Go to question 8.)*

6. What is your tenure status? *(Please select one answer.)*

   Tenured .... ☐ 1
   On the tenure track.. ☐ 2
   Not on the tenure track.. ☐ 3

7. What is the name of your primary employer?

   ____

8. How long have you been at your **current** job?

☐☐year(s) ☐☐ month(s)

9. In what discipline(s) does your current work primarily fall? *(Please select all that apply.)*

Behavioral or social sciences .... ☐[1]
Biological or biomedical sciences....................... ☐[2]
Epidemiology and/or public health .... ☐[3]
Mathematical sciences .... ☐[4]
Medicine.. ☐[5]
Nutrition sciences.. ☐[6]
Physical sciences... ☐[7]
Other *(Please specify.)* .... ☐[8]

____

10. For this question, please exclude time spent on cancer treatment or cancer treatment research. **Approximately** what percentage of your current work is done **in cancer prevention and control?** *(Please select one answer.)*

None .... ☐[1] *(Go to question 11.)*
A small percentage (1%–25%)............................. ☐[2] *(Go to question 12.)*
A moderate percentage (26%–50%) .... ☐[3] *(Go to question 12.)*
A large percentage (51%–75%) .... ☐[4] *(Go to question 12.)*
A very large percentage (76%–100%) .... ☐[5] *(Go to question 12.)*

11. What are the reasons you are **not** currently working in the field of cancer prevention or control? *(Please select all that apply and then go to question 13.)*

A suitable job in the field was not available .... ☐[1]
A better opportunity outside of the field was available.. ☐[2]
My career or professional interests changed .... ☐[3]
Personal reasons.. ☐[4]
Other *(Please specify.)* .... ☐[5]

____

12. **Approximately** what percentage of your time in your current job is spent on **research and research support activities in cancer prevention and control?** Please include time spent conducting research yourself, as well as time spent supporting the research of others through activities such as research management, monitoring, reviewing, funding, analysis, dissemination, and other research support activities. *(Please select one answer.)*

None .... ☐[1]
A small percentage (1%–25%)............................. ☐[2]
A moderate percentage (26%–50%) .... ☐[3]
A large percentage (51%–75%) .... ☐[4]
A very large percentage (76%–100%) .... ☐[5]

13. **Approximately** what percentage of your time in your current job is spent on **all research and research support activities, not just those in cancer prevention and control**? Please include time spent conducting research yourself, as well as time spent supporting the research of others through activities such as research management, monitoring, reviewing, funding, analysis, dissemination, and other research support activities. *(Please select one answer.)*

   - None .... ☐1
   - A small percentage (1%–25%) .... ☐2
   - A moderate percentage (26%–50%) .... ☐3
   - A large percentage (51%–75%) .... ☐4
   - A very large percentage (76%–100%) .... ☐5

14. **Approximately** what percentage of your time in your current job is spent on teaching and advising students? *(Please select one answer.)*

   - None .... ☐1
   - A small percentage (1%–25%) .... ☐2
   - A moderate percentage (26%–50%) .... ☐3
   - A large percentage (51%–75%) .... ☐4
   - A very large percentage (76%–100%) .... ☐5

15. **Multidisciplinary** activities are activities that involve several academic disciplines or professional specializations. To what extent do you currently engage in the following collaborative and multidisciplinary activities with other professionals? *(Please select one answer in each row.)*

**Professional activity**	**Not at all**	**A small extent**	**A moderate extent**	**A large extent**	**A very large extent**
a. Incorporate research from multiple fields/disciplines in your work	☐1	☐2	☐3	☐4	☐5
b. Collaborate with professionals from multiple disciplines	☐1	☐2	☐3	☐4	☐5
c. Manage and/or lead professionals from multiple disciplines	☐1	☐2	☐3	☐4	☐5
d. Present at multidisciplinary conferences or meetings	☐1	☐2	☐3	☐4	☐5
e. Publish in multidisciplinary journals or publications	☐1	☐2	☐3	☐4	☐5
f. Publish with professionals from multiple disciplines	☐1	☐2	☐3	☐4	☐5

CAREER ACTIVITIES

This section asks more detailed information about the types of activities you engage in as part of your work.

16. To what extent have you had a role in the following professional activities **during your career?** *(Please select one answer in each row.)*

Professional activity	Not at all	A small extent	A moderate extent	A large extent	A very large extent	Not applicable
a. Pursued a new theoretical direction or addressed a topic previously unexplored in cancer research........	☐1	☐2	☐3	☐4	☐5	☐6
b. Made a significant contribution to a scientific breakthrough in cancer research	☐1	☐2	☐3	☐4	☐5	☐6
c. Made a significant contribution to advancing innovative ideas in cancer research	☐1	☐2	☐3	☐4	☐5	☐6
d. Addressed key knowledge gaps in cancer research..............................	☐1	☐2	☐3	☐4	☐5	☐6
e. Developed funding initiatives to address knowledge gaps in cancer research	☐1	☐2	☐3	☐4	☐5	☐6

17. For this question, please answer only for activities that occurred after completing your doctoral degree and postdoctoral work, if applicable. **During the past five years,** how many times have you engaged in the following publication activities? *(Please select one answer on each row.)*

Publication activity	None	Once	Two or three times	Four or five times	Six or more times
a. Authored or co-authored a paper in a published peer-reviewed journal.....................	☐1	☐2	☐3	☐4	☐5
b. Authored or co-authored a chapter in a published book..	☐1	☐2	☐3	☐4	☐5
c. Authored or co-authored a published book	☐1	☐2	☐3	☐4	☐5
d. Authored or co-authored a technical report or white paper...	☐1	☐2	☐3	☐4	☐5

18. For this question, please answer only for activities that occurred after completing your doctoral degree and postdoctoral work and exclude presentations given by your students, if applicable. **During the past five years,** how many times have you personally engaged in the following presentation activities? *(Please select one answer on each row.)*

Presentation activity	None	Once	Two or three times	Four or five times	Six or more times
a. Presented at a professional conference or scientific meeting	☐1	☐2	☐3	☐4	☐5
b. Chaired a session or workshop at a professional conference or scientific meeting	☐1	☐2	☐3	☐4	☐5
c. Organized a session or workshop at a professional conference or scientific meeting	☐1	☐2	☐3	☐4	☐5
d. Organized a professional conference or scientific meeting...	☐1	☐2	☐3	☐4	☐5

19. For this question, please answer only for activities that occurred after completing your doctoral degree and postdoctoral work, if applicable. **During the past five years,** how many times have you engaged in the following community service activities? *(Please select one answer on each row.)*

Community service activity	None	Once	Two or three times	Four or five times	Six or more times
a. Advised or presented information to a patient advocacy or support group...................	☐1	☐2	☐3	☐4	☐5
b. Translated cancer research information for a lay audience	☐1	☐2	☐3	☐4	☐5
c. Served on a local health advisory board, panel, or committee	☐1	☐2	☐3	☐4	☐5
d. Served on a national health advisory board, panel, or committee	☐1	☐2	☐3	☐4	☐5

20. For this question, please answer only for activities that occurred after completing your doctoral degree and postdoctoral work, if applicable. During **the past five years,** how many times have you engaged in the following other professional activities? *(Please select one answer on each row.)*

**Other professional activity**	**None**	**Once**	**Two or three times**	**Four or five times**	**Six or more times**
a. Established or appointed to a working group on cancer research	☐ 1	☐ 2	☐ 3	☐ 4	☐ 5
b. Served as a reviewer for a journal	☐ 1	☐ 2	☐ 3	☐ 4	☐ 5
c. Served as an editor of a journal or served on a journal review board	☐ 1	☐ 2	☐ 3	☐ 4	☐ 5
d. Led or co-led a clinical trial............................	☐ 1	☐ 2	☐ 3	☐ 4	☐ 5
e. Received a competitive grant, contract, or subcontract for your work...............................	☐ 1	☐ 2	☐ 3	☐ 4	☐ 5
f. Filed or received a patent	☐ 1	☐ 2	☐ 3	☐ 4	☐ 5
g. Developed a prototype, technology, or marketable product	☐ 1	☐ 2	☐ 3	☐ 4	☐ 5
h. Other professional activity *(Please specify.)* ...	☐ 1	☐ 2	☐ 3	☐ 4	☐ 5

21. What is your current annual salary, including any bonuses you have received? *(Please select one answer.)*

- Less than $50,000 .... ☐ 1
- $50,000–$74,999... ☐ 2
- $75,000–$99,999... ☐ 3
- $100,000–$124,999... ☐ 4
- $125,000–$149,999... ☐ 5
- $150,000–$174,999... ☐ 6
- $175,000–$199,999... ☐ 7
- $200,000–$224,999... ☐ 8
- $225,000 or more .... ☐ 9
- Not applicable .... ☐ 10

22. Do you feel that your salary is generally competitive with others in similar positions?

- Yes... ☐ 1
- No .... ☐ 2
- Not applicable.. ☐ 3

23. Have the following occurred in your career since completing your doctoral degree and postdoctoral work, if applicable? *(Please select one answer on each row.)*

	**Yes**	**No**
Advanced to a more senior-level position	☐1	☐2
Assumed a role as a project leader (e.g., technical group leader)	☐1	☐2
Assumed leadership or management responsibilities (e.g., section chief)	☐1	☐2
Served as a mentor to others in your organization	☐1	☐2
Assumed other leadership roles (e.g., led a committee)	☐1	☐2

24. How satisfied are you with the progression of your career to this point? *(Please select one answer.)*

Not at all	☐1
A little satisfied	☐2
Somewhat satisfied	☐3
Very satisfied	☐4
Extremely satisfied	☐5

PROFESSIONAL ASSOCIATIONS AND AWARDS

This section asks about your participation in professional associations and awards you may have received for your work.

25. How many professional associations (e.g., American Association for Cancer Research, American Medical Association) are you **currently** a member of? *(Please select one answer.)*

None	☐1
One	☐2
Two	☐3
Three	☐4
Four or more	☐5

26. For this question, please answer only for activities that occurred after completing your doctoral degree and postdoctoral work, if applicable. **During the past five years,** have you held either a volunteer or elected leadership position in a professional association? *(Please select one answer on each row.)*

	**Yes**	**No**
A volunteer leadership position	☐1	☐2
An elected leadership position	☐1	☐2

27. For this question, please answer only for activities that occurred after completing your doctoral degree and postdoctoral work, if applicable. **During the past five years,** have you received a professional award related to your work?

Yes *(Please specify.)*... ☐1
No .... ☐2

PROGRAM BENEFITS

This section asks questions about benefits you received from participating in the Cancer Prevention Fellowship Program.

28. **While a fellow,** how beneficial was the Cancer Prevention Fellowship Program to your knowledge, skills, and research in the following areas? *(Please select one answer on each row.)*

Area	Not at all beneficial	A little beneficial	Somewhat beneficial	Very beneficial	Extremely beneficial
a. Scientific subject matter knowledge/expertise..............................	☐1	☐2	☐3	☐4	☐5
b. Knowledge/expertise in public health ...	☐1	☐2	☐3	☐4	☐5
c. Research skills and/or techniques..........	☐1	☐2	☐3	☐4	☐5
d. Experience using specialized equipment and/or technology	☐1	☐2	☐3	☐4	☐5
e. Confidence in performing research	☐1	☐2	☐3	☐4	☐5
f. The overall quality of your research......	☐1	☐2	☐3	☐4	☐5
g. The specific direction of your research ..	☐1	☐2	☐3	☐4	☐5
h. The progress of your research	☐1	☐2	☐3	☐4	☐5
i. Your ability to conduct independent research	☐1	☐2	☐3	☐4	☐5
j. Contacts who advised or collaborated with you on your research......................	☐1	☐2	☐3	☐4	☐5

29. **While a fellow,** how beneficial was the Cancer Prevention Fellowship Program in the following areas pertaining to other professional-related knowledge and skills? *(Please select one answer on each row.)*

Skill	Not at all beneficial	A little beneficial	Somewhat beneficial	Very beneficial	Extremely beneficial
a. Publication skills	☐1	☐2	☐3	☐4	☐5
b. Presentation skills..................................	☐1	☐2	☐3	☐4	☐5
c. Grant and/or contract writing skills........	☐1	☐2	☐3	☐4	☐5
d. Mentoring skills	☐1	☐2	☐3	☐4	☐5
e. Leadership and/or management skills....	☐1	☐2	☐3	☐4	☐5

30. How beneficial was the Cancer Prevention Fellowship Program to you in terms of providing the following career-related benefits? *(Please select one answer on each row.)*

Benefit	Not at all beneficial	A little beneficial	Somewhat beneficial	Very beneficial	Extremely beneficial	Not applicable
a. Securing your first position after the fellowship	☐1	☐2	☐3	☐4	☐5	☐6
b. Securing subsequent positions following your first position after the fellowship	☐1	☐2	☐3	☐4	☐5	☐6
c. Providing you with contacts that have helped you find employment	☐1	☐2	☐3	☐4	☐5	☐6
d. Positively influencing your ability to obtain funding for your work...............................	☐1	☐2	☐3	☐4	☐5	☐6
e. Helping you achieve your career goals	☐1	☐2	☐3	☐4	☐5	☐6
f. Influencing the specific direction of your current research..................................	☐1	☐2	☐3	☐4	☐5	☐6
g. Influencing the progress of your current research	☐1	☐2	☐3	☐4	☐5	☐6

31. Looking back on your career thus far, would you make the same decision to participate in the Cancer Prevention Fellowship Program?

Yes.. ☐1
No .... ☐2

32. Have you ever encouraged someone else to apply for the Cancer Prevention Fellowship Program?

Yes.. ☐1
No .... ☐2

REFLECTIONS AND RECOMMENDATIONS

This section asks a few open-ended questions about significant accomplishments in your career, your opinion about the most valuable aspect of the program, and your recommendations for program improvements.

33. What do you consider to be the two or three most important accomplishments in your career?

34. What was the single most valuable aspect of the Cancer Prevention Fellowship Program to you?

35. What is the single most important improvement you would like made in the Cancer Prevention Fellowship Program?

36. If you could make other improvements to the Cancer Prevention Fellowship Program, what would they be?

DEMOGRAPHICS

The last section of the survey asks about demographic information, including your education.

37. What is your gender?

Male ☐ 1
Female ☐ 2

38. Are you Hispanic/Latino?

No, not Hispanic/Latino ☐ 1
Yes, Hispanic/Latino ☐ 2

39. What is your race? *(Please select all that apply.)*

American Indian or Alaska Native ☐ 1
Asian ☐ 2
Black or African American ☐ 3
Native Hawaiian or Other Pacific Islander ☐ 4
White ☐ 5

40. What is your birth year?

☐☐☐☐

41. Which of the following doctoral degrees have you received and in what year did you receive them? *(Please select all that apply.)*

		**Year of most recent degree**
PhD	☐[1]	________
ScD	☐[2]	________
MD	☐[3]	________
DO	☐[4]	________
DrPH	☐[5]	________
DDS	☐[6]	________
DMD	☐[7]	________
JD	☐[8]	________
DVM	☐[9]	________
Other (*Please specify.*)______________________	☐[10]	________

42. Which of the following other degrees or certifications do you have?

MS	☐[1]
MA	☐[2]
MPH	☐[3]
MBA	☐[4]
NP	☐[5]
RD	☐[6]
RN	☐[7]
Other (*Please specify.)* ____________________	☐[8]
