# Supplementary material for: Independent Association of Postdoctoral Training with Subsequent Careers in Cancer Prevention
Source: PLoS One. 2015 Dec 14;10(12):e0144880. doi: 10.1371/journal.pone.0144880 (PMC4682206; doi:10.1371/journal.pone.0144880)
Supplement: S2 File — (PDF) [file pone.0144880.s002.pdf]

Supplemental File 2 – Description of NRSA/F32 awardee subset and Supplemental Tables A-C.

Supplemental Tables A-C contain analyses from the third population surveyed in this study, NRSA/F32 awardees. These data are an exploratory examination of the association of cancer prevention-related work and research outcomes among another population of individuals who competitively funded their postdoctoral training. The National Institutes of Health (NIH) IMPACII database was used to identify NRSA/F32 postdoctoral fellowship grants awarded by NCI between January 1, 1987 and December 31, 2011.) In an attempt to moderate some of the heterogeneity in research area across the total of 1,543NRSA/F32 recipients during this timeframe, a subset was identified as broadly conducting cancer prevention-related research using the following search terms of NIH grant databases: cancer prevention, vaccine, cancer risk, chemoprevention, antitumor, nutrition, tumor suppressor, carcinogenesis, lifestyle factors, environmental factors, interventions, risk factors, personal behaviors, cancer screening, infections, tobacco, cancer control, occupational factor, early detection, and physical activity. This review initially identified 367 recipients. Of these individuals, five were either deceased or declined the award, leaving a total sampling frame of 362 awardees. Of the 362 F32 awardees, 143 (39%) completed the survey,

Supplemental Table A shows the demographic composition of this group. Overall, this population was more likely to identify with a laboratory-based biomedical research discipline (69%) and work in a university or academic setting (63%). Approximately 24.5% of the NRSA/F32 awardees reported the majority of their current work involving cancer prevention, and among this group 38.8% reported the majority of this work being cancer prevention research-related (Supplemental Table B). Logistic regression analyses demonstrated that

NRSA/F32 awardees were more likely to be conducting cancer prevention related work compared to unsuccessful applicants to the CPFP (OR=4.24) when controlling for other factors (Supplemental Table C).

Table A. Demographics of the NRSA/F32 awardee subset.

	**NRSA/F32 Grant Recipients (%)** N=143
Age (yrs)	
<40	36.4
40-49	47.1
≥50	16.4
Sex	
Male	46.1
Female	53.9
Race/Ethnicity	
White, non-Hispanic	78.9
Other	21.1
Cohort	
1987-1996	12.4
1997-2001	29.0
2002-2006	23.4
2007-2011	35.2
Doctoral Degree	
PhD	94.7
Other	5.3
Discipline[a]	

	**NRSA/F32 Grant Recipients (%)** N=143
Biological science	69.0
Epidemiology[b]	6.2
Behavior/social science	6.2
Medicine	12.4
Nutrition science	0.7
Physical science	11.7
Mathematics	0.7
Other	9.0
No. Disciplines	
One	82.8
More than one	17.2
Employment	
Unemployed	1.38
Currently employed	98.6
Current Employer	
University	62.9
Government: NIH	2.8
Private company	15.4
Government: Other	4.2
Research center	11.2
Self-employed	0.7

	**NRSA/F32 Grant Recipients (%)** N=143
Health clinic/hospital	2.8
Foundation/association	0.0
Years in Current Job	
<5	52.4
5-9	26.6
≥10	21.0

[a] Column percentages >100 because respondents could select more than one discipline
[b] Includes public health
NIH=National Institutes of Health

Table B. Time Spent Performing Cancer Prevention-Related Work or Cancer Prevention Research, by NRSA/F32 awardees.

	**NRSA/F32 Grant Recipients n=140 (%)**
Any Cancer Prevention Work	
None (0%)	30.8
Some (1%-50%)	44.8
Majority (≥51%)	24.5
Cancer Prevention Research[a]	
None (0%)	3.1
Some (1%-50%)	58.2
Majority (≥51%)	38.8

[a] Individuals who answered "None" (n=42) to the question about any cancer prevention-related activities in their current work were not asked the question about cancer prevention research-related activities.

Table C. Odds Ratios for Factors Associated with the Majority of Current Work Involving Cancer Prevention (CPFP applicants and NRSA/F32 awardees).

	**Cancer Prevention Work Odds Ratio (95% CI)**	**Cancer Prevention Research Odds Ratio (95% CI)**
Population Group		
Fellowship applicants	1.00 (referent)	1.00 (referent)
NRSA/F32 grant recipients	4.24 (1.22-14.7)	1.65 (0.47-5.63)
Age (yrs)		
<40	1.00 (referent)	1.00 (referent)
40-49	1.41 (0.50-3.99)	1.44 (0.47-4.39)
≥50	1.74 (0.32-9.41)	0.81 (0.13-5.03)
Sex		
Male	1.00 (referent)	1.00 (referent)
Female	0.71 (0.28-1.80)	1.03 (0.38-2.76)
Race/ethnicity		
White, non-Hispanic	1.00 (referent)	1.00 (referent)
Other than white	0.71 (0.28-1.80)	1.03 (0.38-2.76)
Cohort		
1987-1996	1.00 (referent)	1.00 (referent)
1997-2001	2.13 (0.42-10.9)	2.74 (0.46-16.4)
2002-2006	3.36 (0.50-22.7)	2.33 (0.27-20.2)
2007-2011	5.93 (0.78-44.8)	6.00 (0.63-56.7)
Doctoral Degree		
PhD	1.00 (referent)	1.00 (referent)
Non PhD doctorate	0.32 (0.07-1.49)	0.35 (0.07-1.71)
Epidemiology or Social/Behavioral Science		
No	1.00 (referent)	1.00 (referent)
Yes	14.4 (4.19-49.2)	5.98 (1.67-21.4)

	**Cancer Prevention Work Odds Ratio (95% CI)**	**Cancer Prevention Research Odds Ratio (95% CI)**
Biomedicine		
No	1.00 (referent)	1.00 (referent)
Yes	1.08 (0.40-2.95)	1.24 (0.40-3.88)
Employer		
Other	1.00 (referent)	1.00 (referent)
Government	0.74 (0.13-4.24)	0.30 (0.04-2.04)
University/research center	2.54 (0.75-8.62)	1.35 (0.37-4.94)
No. years in current job		
<5	1.00 (referent)	1.00 (referent)
5-9	1.26 (0.50-3.17)	1.81 (0.67-4.93)
≥10	0.99 (0.24-4.09)	2.10 (0.46-9.62)
